# Common sleep disorders in pregnancy: a review

**DOI:** 10.3389/fmed.2023.1235252

**Published:** 2023-08-21

**Authors:** Allan J. Kember, Praniya Elangainesan, Zachary M. Ferraro, Claire Jones, Sebastian R. Hobson

**Affiliations:** ^1^Department of Obstetrics and Gynaecology, Temerty Faculty of Medicine, University of Toronto, Toronto, ON, Canada; ^2^Dalla Lana School of Public Health, Institute of Health Policy, Management, and Evaluation, University of Toronto, Toronto, ON, Canada; ^3^Shiphrah Biomedical Inc., Toronto, ON, Canada; ^4^Temerty Faculty of Medicine, Medical Education, University of Toronto, Toronto, ON, Canada; ^5^Division of Reproductive Endocrinology and Infertility, Mount Sinai Hospital, Toronto, ON, Canada; ^6^Temerty Faculty of Medicine, Institute of Medical Science, University of Toronto, Toronto, ON, Canada; ^7^Maternal-Fetal Medicine Division, Mount Sinai Hospital, Toronto, ON, Canada

**Keywords:** sleep, pregnancy, reproduction, sleep-disordered breathing, restless legs syndrome, insomnia, circadian rhythm

## Abstract

In this review, we provide a comprehensive overview of common sleep disorders during pregnancy, including their characterization, prevalence, risk factors, and possible contribution to maternal and fetal outcomes. We conducted a quasi-systematic literature search of the MEDLINE database and identified 744 studies from 1991 through 2021, inclusive, that met our inclusion criteria. We synthesized the existing literature on sleep disorders during pregnancy and highlighted controversies, research gaps, and needed clinical developments. Our review covers a range of sleep disorders, including insomnia, obstructive sleep apnea, restless legs syndrome, and circadian rhythm disorders. We discuss the prevalence of these disorders in pregnancy and their potential impact on maternal and fetal health outcomes. We also explore the relationship between sleep disorders, pre-pregnancy comorbidities such as obesity, and pregnancy-related conditions such as gestational diabetes mellitus and preeclampsia. In addition to summarizing the existing literature on sleep disorders during pregnancy, we also highlight opportunities for further research in this area. We suggest that future studies should strive to employ validated and objective measurement tools for sleep disorders and prioritize utilization of longitudinal methods with participant follow-up through postpartum, mid-life, menopause, and beyond. We also put forward investigation into the impact of circadian rhythm disruption on reproductive physiology and early pregnancy outcomes as an area of important work. Overall, our review provides valuable insights on sleep and reproduction and into common sleep disorders during pregnancy and their potential impact on maternal and fetal health outcomes.

## Introduction

Sleep is crucial for human well-being, especially during pregnancy. Evidence indicates that consistently sleeping less than 7 h per night can lead to negative health effects (1). Adequate sleep duration has been extensively linked to positive outcomes in cardiovascular health, cognitive function, mental well-being, physical health, and chronic conditions such as obesity (2, 3). In a recent trial, a 2 weeks sleep extension intervention significantly reduced daily calorie intake in overweight individuals, suggesting that long-term adherence to improved sleep habits could aid in weight management (4). In reproductive-age individuals, studies have examined the effects of shift work, sleep duration, and sleep quality on reproductive and maternal-fetal outcomes (5–7), as well as long-term impacts on child health such as body mass index (BMI) and blood pressure (8, 9).

**Figure 1:**
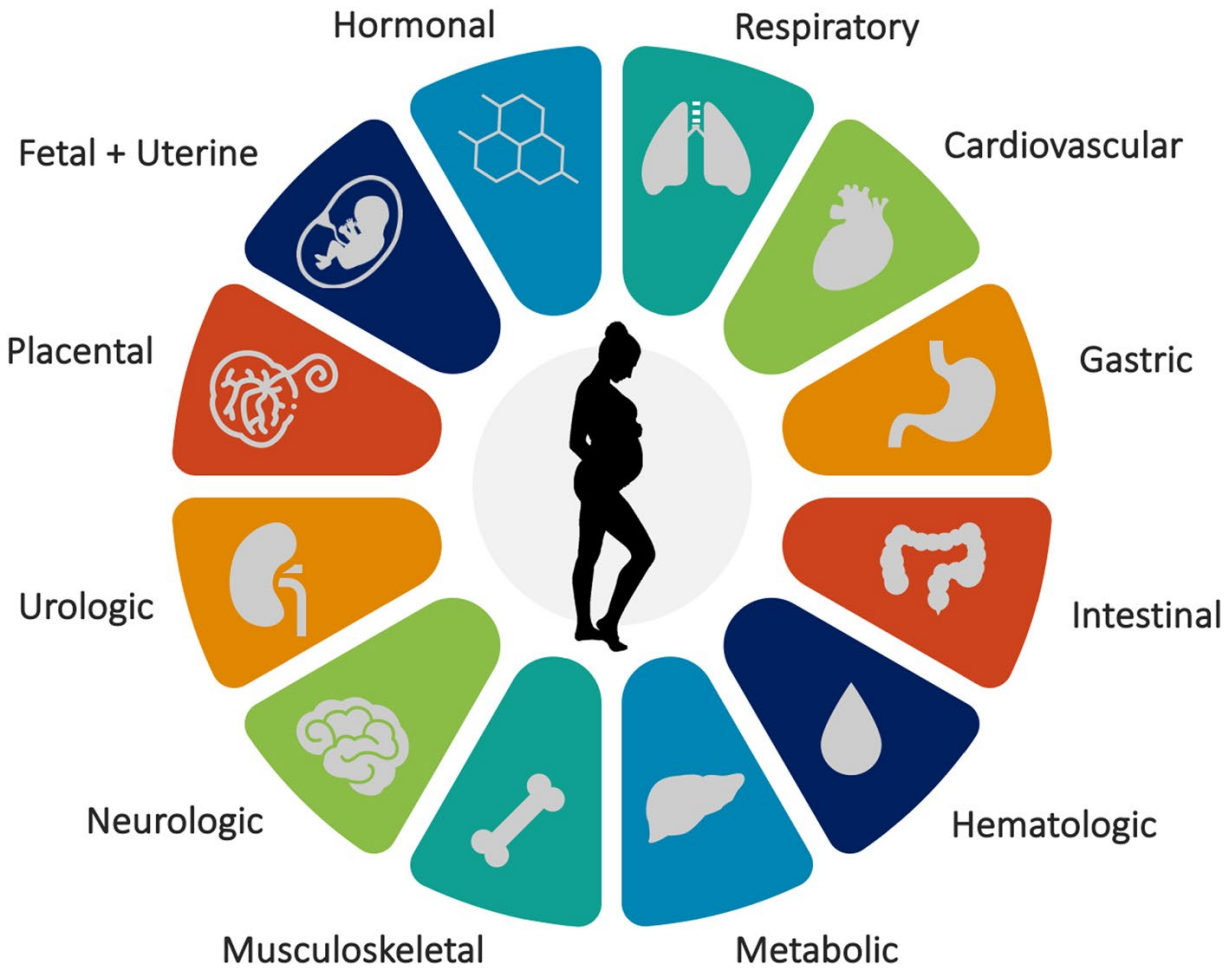
Major organ systems affected by pregnancy.

Pregnancy involves more than fetal growth and organ development—it induces physiological adaptations in nearly all organ systems (Figure 1). Therefore, it is unsurprising that these changes during pregnancy affect and disrupt sleep. Estrogen, progesterone, oxytocin, prolactin, cortisol, and melatonin have dynamic effects on sleep patterns. As a result, pregnancy and sleep intersect to influence the cardiovascular, gastrointestinal, hematologic, metabolic, musculoskeletal, and respiratory systems of pregnant individuals (10–13). The presence of a growing uterus affects bladder capacity, fetal movements, and positional discomfort, leading to frequent awakenings and disrupted sleep quality during pregnancy.

Nearly 50% of pregnant people experience short sleep by term (14). Furthermore, pregnant individuals are more likely to suffer from sleep disturbances and disorders compared to the general population (12, 15). As a general rule, sleep disturbances and disorders in pregnancy are exacerbated by advancing maternal age and gestation, elevated BMI, mental illness, lower educational attainment, and lower socioeconomic status (16–20).

Pregnancy can be impacted by sleep disorders. Insomnia prevalence rises from 25% in the first trimester to over 40% in the third trimester (21, 22). Furthermore, restless legs syndrome (RLS), periodic limb movements, and sleep disordered breathing (SDB) are more common in pregnancy, and circadian disturbances whether organic (e.g., narcolepsy) or related to external factors (e.g., shift work, social, jetlag) continue to play a role in poor sleep (10–13, 18, 21, 23–25). These sleep disorders in pregnancy have well-documented associations with adverse maternal-fetal outcomes. The largest analysis on this topic, conducted by Lu et al. (7), reported on 120 studies with 58,123,250 pregnant people and assessed poor sleep quality, extreme sleep duration, insomnia symptoms, RLS, subjective SDB symptoms, along with obstructive sleep apnea (OSA) and their impact on maternal-fetal outcomes. Their study identified significant associations between sleep disturbances during pregnancy and maternal-fetal complications, including preeclampsia, gestational hypertension (GH), gestational diabetes mellitus (GDM), cesarean section (CS), preterm birth (PTB), large-for-gestational-age (LGA), and stillbirth, but not small-for-gestational-age (SGA) or low birth weight (LBW) (7, 26). However, this study and others (27–36) primarily focus on quantitative estimates of sleep disorders’ effects on maternal-fetal outcomes, neglecting the impact of sleep disorders on future maternal (37), reproductive, and child health.

This review has two main objectives. First, we aim to synthesize the existing literature regarding common sleep disorders during pregnancy including their characterization, prevalence, risk factors, and possible contribution to maternal and fetal outcomes. Second, we aim to initiate academic discourse regarding the influence of circadian disruption on reproductive physiology and early pregnancy outcomes. We will address these objectives and highlight controversies, point out research gaps, and suggest needed research and clinical developments.

## Methods

We conducted a quasi-systematic literature search focused on sleep, reproduction, and pregnancy. The MEDLINE (1946-2021, OVID) database was comprehensively searched on July 20th, 2021, for English articles published between 1991 and 2021 with titles, abstracts, or keywords related to sleep, sleep disorders, pregnancy, reproduction, and fertility.

The inclusion criteria required that the articles: (1) involved individuals planning pregnancy or pregnant individuals, (2) primarily focused on sleep quality, circadian dysrhythmia, sleep quantity, or subjective sleep measures, (3) reported qualitative or quantitative outcomes, and (4) were original research papers or included systematic reviews/meta-analyses. Studies were excluded if they did not report outcomes, if sleep was not an independent variable, or if the full text was unavailable.

**Figure 2:**
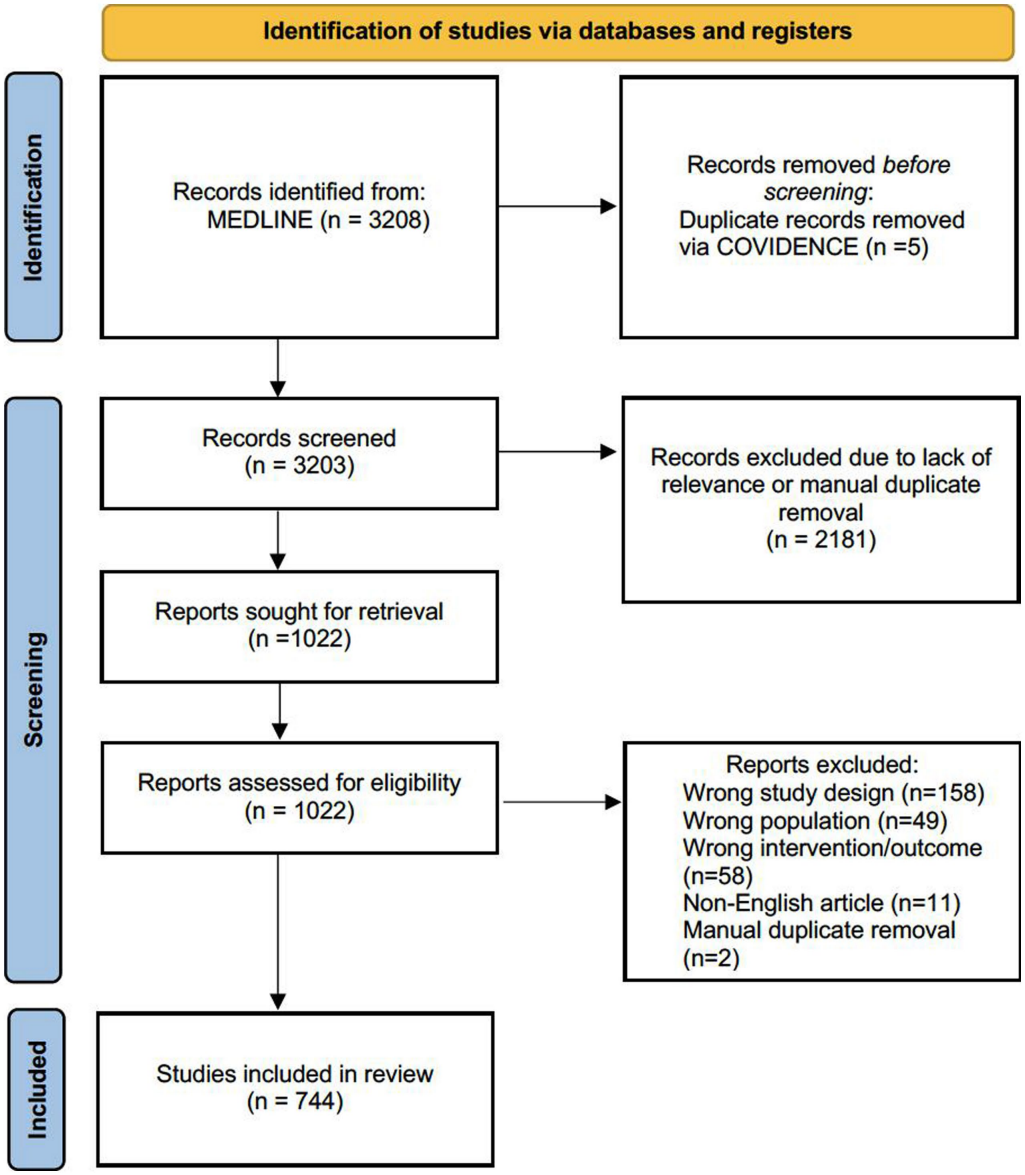
PRISMA flow diagram outlining the study selection process.

Initially, our search yielded 3,208 studies from the MEDLINE database. After removing duplicates and screening titles/abstracts, the number of studies was narrowed down to 1,022. A full-text screening, based on the inclusion criteria, further refined the selection to a final number of 744 studies (Figure 2).

## Sleep-disordered breathing

Our search yielded numerous original research studies and literature reviews on SDB in pregnancy. We found 150 original research articles, nine systematic reviews/meta-analyses, and at least five general literature reviews. Early case studies dating back to 1991 established a link between untreated severe OSA in pregnancy, preeclampsia, fetal heart rate decelerations, maternal blood-oxygen desaturations, and fetal growth restriction (38). Since then, significant progress has been made in SDB research during pregnancy. In the subsequent sections, we will present findings related to the characterization, prevalence, risk factors, and associations of SDB in pregnancy. While subjective measures dominate the literature, some studies employ both subjective and objective measures, while others focus solely on objective measures. Therefore, the following sections generally present subjective research followed by objective research.

### Characterization

There are several ways that SDB can be characterized in pregnancy. Our approach pertains to singleton pregnancies, as studies characterizing SDB in multifetal pregnancies are limited by inadequately powered sample sizes (39). We primarily focus on OSA, which includes a clinical spectrum from snoring to complete airway collapse. Congenital central sleep apnea is rare and has been diagnosed during pregnancy (40). Fortunately, among pregnancies with SDB, central apneas are rare (41). However, at least one study classified more hypopneas as the central type than the obstructive type (42). Given that pregnancy is a transient state impacting and interacting with one’s health, we will review how the characterization of SDB changes across pregnancy and with common pregnancy-associated conditions.

#### Subjective

Pien et al. (43) showed that SDB symptomology undergoes a statistically and clinically significant increase across pregnancy in at least 10% of pregnancies. Self-reported regular apneic episodes (≥3 times/week) peaked in 15% of their respondents at 28–29 weeks, tapering off thereafter to 9% by the final month of pregnancy (43).

#### Objective

Objectively, SDB severity increases across the trimesters and peaks in the initial postpartum period (44, 45). Immediately postpartum, the apnea-hypopnea index (AHI) is significantly higher than it is at 36 weeks (44, 45). Postpartum, the lower levels of progesterone, a respiratory stimulant, may account for this. Lee et al. (46) demonstrated interplay between progesterone levels and OSA by showing that levels in the first and second trimesters, after accounting for weight and gestational age, are significantly lower in pregnancies with OSA. By 3 months postpartum, the AHI and other respiratory metrics significantly improve (47). However, within a given trimester, the literature yields a significant range of AHI values, which is likely due to differing study methodology, namely, study populations and patient comorbidities.

Longitudinal studies are helpful. In a healthy US population, Pien et al. (48) reported a mean AHI of 2.07 events/h in the first trimester, which was significantly higher (3.74) in the third trimester. Maasilta et al. (49) assessed healthy Finnish pregnancies with and without obesity and reported a mean AHI of 0.2 and 0.1 (NS) in pregnancies without obesity in the first and third trimesters, respectively, and 1.7 and 2.6 (NS) in pregnancies with obesity in the first and third trimesters, respectively. Facco et al. (50), studying high-risk pregnancies (BMI ≥30, chronic hypertension, pregestational diabetes, prior preeclampsia, and/or a twin gestation) in a US population, reported their results by AHI category (mild OSA 5 ≤AHI <15; moderate OSA 15 ≤AHI <30; severe OSA AHI ≥30): in the first trimester, 21%, 6%, and 3% of participants had mild, moderate, and severe OSA, respectively, and this rose significantly to 35%, 7%, and 5% in the third trimester, respectively. In high-risk pregnancies, SDB severity worsens as pregnancy advances, particularly for new-onset SDB compared to chronic (pre-pregnancy) SDB. The incidence of new-onset SDB in pregnancy is 1 in 5, with the majority being of mild severity (50).

We found that cross-sectional studies were more common than longitudinal studies. In third-trimester pregnancies without obesity in Israel, Bassan et al. (51) reported a mean AHI of 1.5 and 11.6 in pregnancies without SDB and with SDB, respectively. Sarberg et al. (42) found a median AHI of 0.8 in a cohort of late second-trimester and early third-trimester pregnancies without obesity in Sweden, which was double that found in age- and BMI-matched non-pregnant controls.

Anatomically, in the third trimester, the upper airways are narrower in the seated, supine, and lateral positions compared to non-pregnant controls and postpartum controls (52). Physiologically, Bourjeily et al. (53), in an age-, BMI-, and AHI-matched study, reported that sleeping pregnant people, independent of OSA, have significantly more flow-limited breaths than non-pregnant controls. Prodromakis et al. (44) and Trakada et al. (45) have demonstrated that SDB is characterized by lower mean and minimum maternal arterial partial pressure of oxygen (PaO_2_) levels during sleep and while sleeping supine in the third trimester compared to postpartum regardless of apneas, hypopneas, or percent of REM sleep. Bassan et al. (51), studying peripheral oxygen saturation (SpO_2_), demonstrated a lower mean nocturnal SpO_2_ (94.8%) and lower SpO_2_ nadir (89.8%) in pregnancies with SDB compared to those without (96.1%, and 92.9%, respectively). Sarberg et al. (42) found a lower median SpO2 nadir in their pregnant cohort too. The 4% oxygen desaturation index (ODI4) is the number of events per hour of sleep in which the SpO_2_ drops by 4% or more. In the first trimester of pregnancy, Maasilta et al. (49) reported an ODI4 of 0.3 and 5.3 for pregnancies without and with obesity, respectively, and in the third trimester, these values increased to 0.5 and 8.9. Bassan et al. (51) reported a mean ODI4 of 2.9 and 0.3 in third-trimester pregnancies with and without SDB, respectively. Sarberg et al. (42) reported a median ODI4 of 0.5 and 0.2 in their pregnant and matched non-pregnant cohorts, respectively.

Biochemically, according to Khan et al. (54), pregnancies with OSA appear to have a higher antioxidant capacity and significantly lower carbonyl stress markers in the second trimester compared to pregnancies without OSA. However, treatment with continuous positive airway pressure (CPAP, known to reduce oxidative and carbonyl stress) was not documented, and OSA was not definitively excluded from the controls. Indeed, the opposite finding is well established in the non-pregnant literature, so caution is warranted.

In summary, the characterization of OSA in pregnancy represents an interplay between two complex and dynamic physiologies—OSA physiology and pregnancy physiology—with both contributory and resultant anatomical, physiological, and biochemical alterations. The severity of OSA worsens with advancing gestation and peaks in the immediate postpartum period, and most OSA in pregnancy is in the mild category.

Particular to SDB characterization, two pregnancy conditions—gestational diabetes mellitus (GDM) and hypertensive disorders of pregnancy (HDP)—warrant separate consideration, which we give here. The reader should note that in the SDB prevalence section, we will also consider these conditions separately, along with obesity.

##### Gestational diabetes mellitus

Insulin resistance is a natural aspect of pregnancy, ensuring an adequate glucose supply to the developing fetus. However, when maternal pancreatic function cannot overcome this insulin resistance, aberrant glucose control ensues and results in elevated maternal blood glucose levels and a diagnosis of gestational diabetes mellitus (GDM). Reutrakul et al. (55) investigated pregnancies late pregnancy and demonstrated a median AHI of 8.2, 2.0, and 0.5 in pregnancies with GDM, pregnancies without GDM, and non-pregnant controls with normal glucose tolerance, respectively. Bublitz et al. (56) found lower cortisol awakening responses in pregnancies with GDM that spend a greater amount of time with SpO_2_ below 90% overnight, and they found an attenuated cortisol awakening response in pregnancies with AHI ≥5.

##### Hypertensive disorders of pregnancy

Elevated blood pressure first presenting after 20 weeks’ gestation broadly categorizes one as hypertensive in pregnancy. Compared to GDM, we found a greater focus on hypertensive disorders of pregnancy (HDP). In a multiethnic, non-obese cohort, Champagne et al. (57) reported mean AHI’s of 18.2 and 38.6 in their normotensive and GHTN groups, respectively, and mean ODI4’s of 0.2 and 4.0 in the same groups, respectively. In Iran, Keshavarzi et al. (58) reported mean AHI’s of 17 and 22.5 in their normotensive and preeclampsia groups, respectively, and a higher prevalence of severe OSA in their preeclamptic group. In a US population with HDP, O’Brien et al. (59) reported a mean AHI of 3.4 and 19.9 in non-snorers and snorers, respectively, and a mean SpO_2_ nadir of 90.3% and 86.4% in the same groups, respectively. Only the snorers had SpO_2_ desaturations to less than 80%.

During an episode of nocturnal airway obstruction of any clinical severity, Reid et al. (60) reported a significant transient increase in systolic and diastolic blood pressure of about 30 and 15 mmHg, respectively, and this held in pregnancies with and without gestational hypertension. In preeclampsia, Edwards et al. (61) demonstrated that the pressor response to obstructive respiratory events is markedly augmented but without differences in heart rate responses compared to controls. In preeclampsia, a greater proportion of sleep time is spent with inspiratory airflow limitation, lasting several minutes and without associated oxygen desaturations, compared to normotensive pregnancies (62, 63). Blyton et al. (64) corroborated this but found increased oxygen desaturations, particularly during REM sleep, in their preeclamptic group. Overall, the evidence suggests that airway obstruction during pregnancy has the ability to modify hemodynamics independent of an underlying HDP diagnosis.

### Prevalence

Our review revealed significant variation in the prevalence of SDB, which was influenced by study population, design, gestational age at assessment, presence of comorbidities, and subjective or objective measurement methods. Incongruence between subjective and objective measures of SDB in pregnancy has been documented (42), leading us to present them separately.

#### Subjective

Habitual snoring has been defined variably: snoring for three or more nights per week (52, 59, 65–69), more conservatively (70–73), by frequency category (e.g., “often,” “frequently,” “almost always,” “always,” “most of the time,” “all of the time”) (74–78), by simply the presence/absence of snoring (79, 80), or not based on snoring frequency at all (42). Variability in definitions results in variability in reported prevalence. In one study reporting prevalence in the third trimester as “any frequency of snoring or choking/gasping” separately from “snoring three or more nights per week or choking/gasping,” they gave 63.9% for the former definition and 42.0% for the latter (66).

Few studies delineate between pregnancy-onset and chronic snoring. In these studies, the prevalence of pregnancy-onset snoring is between 11.7%–28% in the third trimester (71, 81–83) and up to 49% in preeclampsia (83). In the third trimester, the prevalence of chronic snoring is reported as 4.9%–27% (71, 81, 82) and up to 36% in preeclampsia (83). Dunietz et al. (84) examined the frequency and severity of snoring in a US population of non-hypertensive, non-diabetic pregnancies in the third trimester: most of the pregnancy-onset snoring had onset in the second trimester. For infrequent quiet snoring, the prevalence of pregnancy-onset snoring and chronic snoring was 64% and 41%, respectively. For frequent quiet snoring, these values were 29% and 5%, respectively, and for frequent loud snoring, 7% and 4%, respectively.

Longitudinal studies report snoring prevalence as 6.7%–29.7% in the first trimester (42, 65, 72, 85), 8.1%–40.5% in the second trimester (65, 72, 85), and 13.0%–46.2% in the third trimester (42, 65, 72, 85). Point prevalence studies report snoring prevalence as 7.3%–13% in the first trimester (77, 78), 39.3% in the second trimester (73), and 11.9%–42% (with most studies reporting in the 30%–40% range) in the third trimester (52, 59, 66–70, 74–76, 79). Further, studies using a conservative definition report snoring prevalence as low as 2.5% in the third trimester (76), whereas those using a less conservative definition report up to 63.9% (66).

Regarding comorbidities, in the USA, Olivarez et al. (86) reported a prevalence of “high risk for OSA” of 90% in first-trimester pregnancies with BMI ≥30 and 8.9% in those with BMI <30. In China, Leung et al. (72) reported the prevalence of moderate (3–4 nights/week) to severe (5–7 nights/week) snoring as 20.8% in third-trimester pregnancies with BMI ≥25 compared to 5.3% in those with BMI <25. In HDP, O’Brien et al. (59) found a 61% prevalence of habitual snoring in the third trimester compared to 19% in normotensive controls. Similarly, Karaduman et al. (87) found a higher all-trimesters prevalence of “high risk for OSA” in pregnancies with chronic diseases (45.4%) compared to healthy pregnancies (10%).

Other studies have reported prevalence of being “high risk for OSA” based on screening tools such as the Berlin questionnaire (BQ) or the Epworth sleepiness scale (ESS) on the order of 29%–56%, which we will not present in further detail here (85, 88–90). Witnessed apneas in pregnancy are studied less frequently. In the first, second, and third trimesters, prevalences have been reported as 0.3%–0.4%, 0.3%–0.4%, and 0.4%–1.1%, respectively (65, 76, 85), compared to 0.2% in non-pregnant controls (85).

In sum, the prevalence of subjectively-reported snoring has wide variability and is increased by advancing gestation and the presence of comorbidities.

#### Objective

While objectively-assessed SDB may give a more accurate picture of prevalence, we found significant variability in timing of assessments, reporting measures, and patient populations, which warrants careful attention.

Sarberg et al. (42) reported a median of 9% of total sleep time spent snoring in the third trimester compared to 4% in age- and BMI-matched non-pregnant controls. Maasilta et al. (49) found objectively-measured snoring prevalence to be 1.1% and 32.0% in the first trimester in pregnancies without and with obesity, respectively, and 1.2% and 48.8% in the third trimester in pregnancies without and with obesity, respectively.

The most common definition of OSA is AHI ≥5. We use this definition unless otherwise stated. The worldwide prevalence of OSA across all three trimesters was reported at 19% (95% CI: 10%–28%) with highest prevalence in the Americas region (20%) and lowest in the European region (5%) (31). In the first trimester, OSA prevalence is between 3.6%–10.5% (48, 91). We found no second-trimester studies of OSA prevalence; however, in the second half of the second trimester and the third trimester, OSA prevalence is 3%–16.1% (42, 91, 92). In studies of the third trimester alone, OSA prevalence is 25%–37% (48, 51, 93, 94). Database studies report increasing year-over-year OSA prevalence in pregnancy and estimate the overall rate around 3.0–8.7 per 10,000 (95, 96). Reliant on diagnostic codes, this may be a significant underestimate of prevalence.

##### Obesity

The prevalence of OSA is higher in pregnancies affected by obesity. In the late second trimester and early third trimester, OSA prevalence is 15.4%–43.3% (97–99). At closer look, 81%–86% of these were cases of mild- to moderate-OSA and 14%–19% were severe-OSA (98, 99). In third-trimester studies, OSA prevalence is 37.5%–67% (100, 101).

##### Gestational diabetes mellitus

The prevalence of OSA is higher in pregnancies affected by GDM. In the late second trimester or early third trimester, Reutrakul et al. (55) reported a 73% prevalence of OSA in those with GDM compared to 27% in those without. Wanitcharoenkul’s et al. (102) results are corroborative, reporting an OSA prevalence of 52.4% in pregnancies with diet-controlled GDM. The OSA was mild, moderate, and severe in 81.3%, 16.3%, and 2.3% of Wanitcharoenkul’s et al. (102) cases, respectively. Assessing OSA at a mean of 28–29 weeks’ gestation in pregnancies with GDM, prevalences are reported as low as 17% (56) and as high as 66% (this study’s threshold for OSA was AHI ≥10) (103). While BMI is a well-known and independent predictor of OSA, when the relationship between OSA and GDM is adjusted for BMI, the association remains significant (36, 55).

##### Hypertensive disorders of pregnancy

The prevalence of OSA is also higher in pregnancies with hypertensive disorders of pregnancy (HDP), ranging from 41%–53% compared to 12%–19% in normotensive pregnancies (59, 104). O’Brien et al. (59), investigating a third-trimester, US-based population, reported that approximately 1 in 10 with HDP have severe OSA, and approximately 1 in 4 with HDP *and* snoring have moderate to severe OSA. Wilson et al. (101) found no difference in OSA prevalence between third-trimester HDP cases (52.5%) and BMI- and gestational-age-matched normotensive controls (37.5%), demonstrating the importance of BMI as a covariate. However, when severity was considered, the cases had more than double (35% vs. 15%) the prevalence of severe-OSA compared to their matched controls.

### Risk factors

We divide studies reporting risk factors for SDB in pregnancy as those that used subjective measures and those that used objective measures because screening questionnaires (e.g., BQ) have low sensitivity and specificity for clinical use in pregnancy. A recent study demonstrated that, in pregnancy, positivity on the BQ is predominantly a proxy for obesity, not SDB (105).

#### Subjective

Risk factors for self-reported snoring, “high risk for OSA” on BQ, and higher apnea symptom scores include increasing and elevated BMI (42, 80, 82, 88, 106, 107), including pre-pregnancy overweight and obesity (43, 49, 73, 74, 78, 108, 109), and gestational weight gain (GWG) (76, 88); anthropometric variables such as larger increases in neck (43, 85) and waist circumference during pregnancy (85); habitual snoring prior to pregnancy (78); advanced maternal age (≥35 years) (74, 78); maternal smoking (74, 78, 85, 109) and alcohol use; and history of allergic rhinitis or asthma (85), pregestational diabetes (78), mood and anxiety disorders (78), pre-pregnancy medical disorder (108), HDP (59), and family history of diabetes (109). Negative correlations include tonsillectomy, higher socioeconomic status, living in rural areas, and higher education (85). While the majority of the literature agrees on these risk factors, a Swedish study did not find an association between GWG nor maternal age and apnea symptom score and self-reported snoring (42).

#### Objective

Risk factors for objectively-verified SDB are similar. Higher pre-pregnancy BMI (51), higher first trimester BMI (48), and obesity are risk factors for OSA in pregnancy (33, 49, 98, 99, 110). In pregnancies with GDM, higher BMI in the third trimester and greater GWG are risk factors for increased OSA severity independent of glycemic control (103). In HDP, after stratification for obesity, self-reported snoring is a risk factor for OSA, doubling its odds (59). Increasing neck circumference (51, 110) and maternal age are risk factors for OSA (33, 48, 99). The literature also implicates asthma (110), pregestational diabetes mellitus (110), and chronic hypertension as OSA risk factors (98, 99, 110).

### Associations/outcomes

In pregnancy, the literature often separates the mother from the fetus, and we adhere to this division in our review of associations. We discuss maternal associations with SDB and fetal associations with maternal SDB, reviewing subjective and objective measures in each sub-section, where applicable.

#### Maternal

##### Subjective SDB

Preeclampsia is associated with habitual snoring (74, 111, 112), any snoring (75), pregnancy-onset snoring (113), “high risk of OSA” (BQ) (73, 89, 108), positive BQ or ESS in the first trimester (86), and self-reported symptoms of SDB (69). Systolic, diastolic, and mean arterial blood pressure are positively correlated with self-reported snoring (79). Gestational hypertension (GH) is associated with witnessed apneas (75), habitual snoring (74, 111), habitual snoring only when comorbid with insomnia (67), increasing nightly frequency of snoring (114), pregnancy-onset snoring (113), positive BQ or ESS in the first trimester (115), and self-reported symptoms of SDB (69). Few studies have contradictory findings to the above (76, 88). A causal mediation analysis by Dunietz et al. (116) lends insight: pregnancy-onset snoring in those without pre-pregnancy hypertension significantly mediates (15%) the relationship between excessive weight and HDP.

Associations between GDM and subjective SDB are nuanced. GDM is associated with self-reported symptoms of SDB (69), positive BQ and/or ESS in the first trimester (108), and chronic snoring (82). Qiu et al. (109) demonstrated an association between GDM and impaired glucose tolerance and frequent snoring (“most or all of the time”) in early pregnancy, which remained significant after adjusting for confounders. They highlighted an interaction effect of pre-pregnancy BMI: compared to lean patients who did not snore, lean patients who snored had a two-fold higher odds of GDM, and overweight patients who snored had a five-fold higher odds of GDM. They found a direct relationship between frequency of snoring and oral glucose tolerance test results, but when accounting for pre-pregnancy BMI, this only remained significant for the highest snoring frequency category (109). Associations have also been reported between hyperglycemia and habitual snoring (77), loud snoring, snorting/gasping, and apneas (77). Again, few studies have contradictory findings (113).

Cesarean birth is associated with chronic snoring (81), pregnancy-onset snoring (81, 82), increasing nightly frequency of snoring (114), and “high risk of OSA” (BQ) (88). Unplanned and emergency cesarean birth is associated with symptoms of SDB (69), pregnancy-onset snoring (81), and habitual snoring (≥3 nights/week) in the third trimester (67).

With regard to other pathophysiology in pregnancy, subjectively-reported SDB is also associated with excessive daytime sleepiness (42, 43, 67, 75), depressive symptoms (68), sympathetic predominance in resting cardiac autonomic tone (117), edema in late pregnancy (42), placental adhesion (82), and alterations in biomarkers that indicate lower reserve for antioxidant defense, a shift toward oxidation, and a state of higher oxidative stress but similar antioxidant activity (118, 119). Maternal serum placental biomarker levels in the first trimester (PAPP-A) and second trimester (AFP, uE3, hCG, inhibin-A) were not associated with chronic nor pregnancy-onset snoring in one study (120).

Regarding long-term impacts of subjectively-determined SDB on maternal and offspring health, Chaggar et al. (121) reported that snoring in pregnancy was not associated with OSA later in life; however, their study was limited by self-reporting, recall bias (~30 years between pregnancy and self-report), and a low prevalence (2.9%) of snoring in pregnancy. More work is needed to elucidate the prognostic implications of SDB in pregnancy on long-term health (37).

##### Objective SDB

The literature on objectively-assessed OSA in pregnancy generally aligns with subjective assessments but provides additional insights.

An association between eclampsia and OSA has been reported (96, 122). The association between preeclampsia and OSA is well established with odds ratios (OR’s) and adjusted OR’s (aOR’s) in the range of 1.6 to 3.5 (31, 32, 34, 95, 96, 110, 122–124). This has been confirmed with OSA occurring in early pregnancy or late pregnancy (91) and particularly in the moderate- to severe-OSA categories (35). Historically, OSA has been thought to exacerbate preeclampsia *via* worsening endothelial dysfunction (125). Recent work challenging these findings highlights the need for an additional level of scrutiny. In one study of high-risk pregnancies, no association was found between preeclampsia and early- or late-onset OSA in pregnancy (126). Furthermore, in pregnancies affected by HDP, being co-diagnosed with OSA does not worsen maternal outcome when compared to no OSA, and no difference between severity of hypertension, anti-hypertensive treatment, and biochemical, hematological and anti-angiogenic markers was found in those with OSA compared to those without (127).

The OR’s and aOR’s of OSA for GH are reported from 1.43 to 7.5 (31, 32, 57, 95, 110, 128). While the aOR of OSA in early pregnancy for GH did not reach statistical significance, it did for mid-pregnancy in a high-quality study by Facco (aOR 1.73) (91). Furthermore, moderate-to severe-range OSA has been associated with GH in a meta-analysis (35).

An association between GDM and OSA is well-established (31, 32, 91, 99, 122) and remains significant when adjusted for BMI (36, 55). More frequent and more severe OSA events are associated with GDM (35, 129) and higher fasting glucose levels (35, 102). In pregnancies with GDM, Newbold et al. (103) demonstrated that increasing AHI is independently associated with elevations in nighttime, morning, and hourly glucose levels. Corroborating these results are Wanitcharoenkul et al. (102) and Farabi et al. (100) In early pregnancy, Facco et al. (91, 126) demonstrated that OSA is associated with GDM (aOR’s 3.5–3.6), and GDM risk is higher with increasing severity of OSA in high-risk pregnancies (126). That said, Izci Balserak et al. (77) found no association between AHI in the first trimester and glucose challenge test results. Late second and early third trimester OSA is associated with GDM (55, 91). Studies of potential mechanisms underlying the association between OSA in pregnancy and GDM are few (130). Furthermore, in some studies, including a meta-analysis (34), the association between OSA and GDM did not reach statistical significance (128, 131).

Again, OSA is associated with cesarean birth. The aORs range from 1.42 to 1.73 (31, 33, 34, 95, 98, 124) [OR 1.38 for elective CS (33), and OR 2.52 for emergency CS (33)], with one study showing no association (128). Other peripartum associations include assisted vaginal delivery (OR 1.88) (33), postoperative wound complication (OR 3.67, aOR 1.87) (31, 33), pulmonary edema (aOR 6.35) (31), pulmonary hypertension (case report) (132), pulmonary embolism (aOR 4.5) (96), cardiomyopathy, congestive heart failure, and hysterectomy [OR’s 2.5–3.5, aOR 9.0 for cardiomyopathy (96)] (122), ICU admission (aOR 2.74) (122), significantly longer hospital stay [while others showed association (31)] (122), in-hospital mortality (aOR 5.28) (96), and decreased resting heart rate variability (133). Facco et al. (134) observed an exposure-response relationship between OSA severity and a composite measure (HDP, GDM, or PTB ≤34 weeks): pregnancies with mild-, moderate, and severe-OSA had an 18.1, 23.5%, and 38.5% prevalence of the composite. Redhead et al. (92) reported an association between OSA and depression symptoms (aOR 8.36), especially in those with a history of depression. Biochemically, OSA is associated with higher CRP and decreased HDL-cholesterol (135), decreased MoM level of PAPP-A (136), increased ratio of VEGF:PLGF (136), and lower estriol level (137).

#### Fetal

In this section, a recent and monumental contribution by Warland et al. (26) deserves attention and discussion. For birth weight, fetal growth, PTB, and stillbirth, which are the four fetal outcomes that Warland et al. considered, we will summarize their findings and then add any additional studies and associations that our search uncovered.

##### Subjective SDB

Warland et al. (26) found no association between subjective SDB, birth weight, and fetal growth; however, five of the largest studies (≥1,000 participants) have conflicting results (26): three in favor of an association between chronic snoring and SGA (69, 81, 138), and one in favor of an association between a pregnancy-onset snoring and macrosomia and LGA (82). Our findings agree, and we found only one additional study (due to recency of publication) where self-reported habitual snoring (≥3 nights/week) was associated with LGA (*n* = 439, aOR 3.5) (67). Warland et al. (26) found that the relationship between subjective SDB and PTB was considered in only eleven studies, is inconsistent, but is in favor of an association in four of the five largest studies. Studies considering stillbirth and subjective SDB are few, underpowered given the relative low prevalence of stillbirth in high-income countries, and limited by potential recall bias (26).

Habitual snoring in pregnancy is associated with Apgar scores ≤7 at 1 and 5 min (111). Symptoms of SDB are associated with poor neonatal outcomes (aOR 2.77), emergency operative birth (instrumental, aOR 2.81; instrumental or CS, aOR 2.32) and an operative birth for intrapartum fetal compromise (aOR 2.62) (139). Dunietz et al. (84) found that chronic frequent-loud snoring is associated with increased hazard for earlier deliveries (adjusted HR 1.60): 24% of chronic frequent-loud snorers (absent of key pregnancy comorbidities) delivered before the completion of 37 weeks’ gestation compared to only 10% of the non-snorers. Pregnancy-onset snoring frequency and intensity was not associated with time-to-delivery (84).

Compared with non-snorers, habitual snoring in pregnancy is associated with significantly elevated cord blood levels of circulating nucleated fetal red blood cells, erythropoietin, and IL-6—all of which point to fetal hypoxemia (for a duration of at least 28–29 h) (140) and/or maternal diabetes (70). Symptoms of SDB during pregnancy are associated with shortened telomere length, indicating a possible role in accelerated chromosomal aging (141). The scarcity of biomarker studies make this an interesting area of future investigation.

Some studies found no association between snoring and fetal outcomes (76, 142). In one, of 11 patients who self-reported frequent apnea, four had overnight in-lab polysomnography (PSG) of which two were diagnosed with mild OSA and one with positional OSA, highlighting the importance of objective measurements (142). Furthermore, Robertson et al. (66) did not observe an association between SDB symptoms in the third trimester and feto-placental Dopplers, fetal cardiac function parameters, or fetal regional cerebral blood flow.

##### Objective SDB

Warland et al. (26) found fewer (*n* = 16) studies using objective measures OSA. Literature regarding objective OSA, birthweight, and fetal growth was limited and inconsistent; however, the largest prospective study of objective OSA in association with fetal growth (*N* = 230) found a 2–3 fold increase in SGA with increasing OSA severity (OR 2.65). Contextualized, two studies demonstrating a fall across fetal growth centiles (>33% in one study) when fetal growth is assessed serially across the third trimester in pregnancies with OSA lend support to the idea that OSA impacts fetal growth regardless of whether growth is affected enough to be classified as SGA or FGR (90, 143).

Since Warland’s et al. metaanalysis, Brown’s et al. (33) systematic review and metaanalysis reported an unadjusted OR of 3.57 (NS, *p* = 0.12) of OSA for LBW. They also reported an aOR (adjusted for BMI and age) of 1.54 (*p* = 0.001) of SDB for SGA based on three studies using objective measures and one study using subjective measures (33). Liu’s et al. (31) systematic review and metaanalysis did not find an association between mid-pregnancy SDB and SGA. Even more recently, Kidron et al. (144) demonstrated that the birthweight-to-placenta ratio is significantly lower in pregnancies with OSA and placenta weight is significantly increased. Wilson et al. (145) found that mild SDB did not adversely affect fetal growth or size at birth in pregnancies with HDP nor in normotensive pregnancies. Brenner et al. (135) demonstrated that offspring of mothers with SDB in pregnancy had a significantly smaller head circumference at birth and compromised birth weight-to-length followed by rapid catch up growth and an increase in both weight-to-length and tricep thickness by age three. In another study not captured by any metaanalysis to date, Hawkins et al. (146) did not find an association between SDB in early- or mid-pregnancy, SGA, or LGA; however, they did report an association between some measures nocturnal hypoxemia and LGA, independent of BMI. This association with LGA is corroborated by another recent study by Telerant et al. (147).

Of thirteen studies looking at objective OSA and PTB considered by Warland et al., significant associations between OSA and PTB that were found in four larger studies (95, 124, 128, 148) were not confirmed in seven smaller (all *N* < 230) studies (49, 51, 98, 107, 126, 134, 149); however, all studies had serious limitations that made it difficult to draw any conclusions. Liu’s et al. (31) metaanalysis reviewed seven studies and gave an aOR of 1.62 of OSA for PTB. Brown’s et al. (33) metaanalysis reviewed six studies and gave an aOR of 2.00 of OSA for PTB. All the studies included in Liu’s et al. and Brown’s et al. analyses were included in Warland’s et al. Warland et al. included several additional studies for the PTB outcome *via* a less strict inclusion criteria [e.g., Liu’s et al. review excluded studies that included participants with comorbidities, such as Facco et al. (126)] and by completing calculations using raw data.

Warland et al. found only three studies investigating OSA and stillbirth, and no conclusions could be drawn due to lack of sufficient data (96, 98, 128, 150). Brown’s et al. (33) metaanalysis did find an association between OSA and stillbirth and/or perinatal death (OR 2.02). This discrepancy is likely because Brown et al. combined stillbirth and perinatal death into one metric, analyzing the same three studies that Warland et al. analyzed along with three additional studies (Leung et al. (72) and Owusu et al. (112), which used subjective measures of OSA; and Spence et al. (95), which used objective measures).

Two studies have shown fetal heart rate (FHR) decelerations accompanying maternal oxygen desaturations (90, 151), whereas another did not show an association between the FHR, OSA parameters, or apnea episodes (107). Wilson et al. (145) found that mild SDB in pregnancies with known FGR did not exacerbate FHR decelerations despite a higher prevalence of FHR decelerations in the pregnancies with FGR. However, there was an independent association between mild-type nocturnal FHR events and AHI, and this was mainly seen in normotensive patients. A major limitation is that these studies did not report maternal sleeping position, which is a known confounder. A 1996 study by Loube et al. (142) is the only study, in over two-and-a-half decades, of position-dependency of OSA in pregnancy that we are aware of. Only recently, Wilson et al. (152) accounted for the position-dependency of OSA, demonstrating a 43% prevalence in the third trimester. Positional dependency (supine AHI is more than twice the non-supine AHI) is usually lost in patients whose BMI class is overweight or obese or in patients who have recently gained significant weight (153), which is usually the case in mid to late pregnancy. As such, the moderately high prevalence of positional OSA into the third trimester seems counter-intuitive and an interesting avenue of future work.

There are associations between OSA and requirement for neonatal resuscitation (aOR 2.76) (154), lower Apgar scores (151), including 5 min Apgar <7 [OR 2.14 (33), aOR 1.60 (128)], NICU admission (OR 1.9, aOR 1.26–2.65) (31, 33, 34, 98, 128, 151, 154), and longer length of hospital stay for the neonate (aOR 2.25) (154). Moderate-to severe-OSA is associated with 1 min Apgar <7 (OR 1.78) (35) and NICU admission (OR 2.43) (35). Contrariwise, one metaanalysis found no association between OSA and acidosis at birth nor meconium stained amniotic fluid (33), and another study found no association between OSA and 5 min Apgar score (51), but the mean AHI in that study was in the mild range.

Maternal OSA is also associated with a higher risk of congenital anomalies in offspring (aOR 1.26), particularly musculoskeletal anomalies (aOR 1.89) (154). It is also associated with low reading test scores (aRR 1.55) (150), low social developmental score at 12 months (aOR 16.7) (94), infant snoring (94), and significantly increased hospitalizations in the first year of life (aHR 1.81) and between the first and sixth birthdays (aHR 1.41) partially due to admissions for suspected pediatric sleep apnea (150). Furthermore, OSA and histopathologic evidence of chronic fetoplacental hypoxia, as manifested by fetal normoblastemia and increased placental carbonic anhydrase IX immunoreactivity has been reported (155). Biochemically, a non-significant trend of lower IGF-1 and higher IGFBP-1 and IGFBP-2 in the cord blood of infants of pregnancies with OSA was reported in an Australian population (90).

Studies reporting no association between OSA and outcomes included developmental vulnerability (scoring below the 10th percentile of the national population), special needs status, and low numeracy test scores (150), gestational age at birth, and neonatal neurologic examination scores within 48 h of birth (51). Furthermore, a lack of an association between OSA and infant general movements assessed at the first 48 h, at 8–11 weeks, and 14–16 weeks (94), and uteroplacental underperfusion scores have also been reported (155).

## Restless legs syndrome

Restless legs syndrome (RLS) or Willis–Ekbom disease, is a common sleep and movement disorder in pregnancy which has been well-studied. Our search for this disorder yielded 42 studies.

### Characterization

RLS manifests as an overwhelming urge to move one’s legs due to unpleasant sensations or dysesthesia (156). The pathophysiology of RLS is not completely understood, however genetic predisposition, central nervous system iron deficiency, and dopaminergic dysfunction have been implicated (157). In pregnancy, hormonal alterations and imbalance related to reproduction may explain transient RLS. Specifically, estradiol-mediated dopamine overmodulation and subsequent decreased inhibition of thyrotropin causing increased thyroid stimulating hormone may lead to that imbalance between the two hormones precipitating RLS (158). Thus, pregnancy is a common cause of secondary RLS with symptoms typically beginning during pregnancy or after childbirth (159). Symptoms are most common during the second and third trimester with resolution post-delivery, while some studies have shown resolution within 2 weeks postpartum (124, 160–162). The sensations in particularly more commonly affect the calves compared to both the calves and thighs (163). Body mass may also play a role in the pathophysiology as mean weight gain is significantly higher in women with RLS compared to those without RLS (164).

Several studies have shown that people with transient RLS in pregnancy commonly experience moderate to severe symptoms (160, 161, 165). In a cohort of 2,900 pregnant people, 49.1% experienced severe or very severe RLS (165). Sleep quality deteriorates in pregnant people with more severe RLS symptoms (164). Overall, pregnant people with RLS were found to have poorer quality of life in terms of physical functioning, physical role limitations, pain, general health perception, energy/vitality, and mental health (165).

### Prevalence

The prevalence of RLS in pregnancy was between 0 to 46.4% with significant variability across different populations (15, 23, 166–173). In Chen’s et al. (15) meta-analysis of 27 articles, they concluded that the pooled prevalence of RLS across all trimesters of pregnancy was 21%. Using the definition of jumpy or jerky leg movements for RLS, the prevalence was slightly higher at 35.5% (174). A common consensus is that the prevalence peaks within the third trimester (15, 23, 167, 168, 170). This was consistent within a meta-analysis of 10 studies that found the total prevalence in the third trimester to be 22.9% (23).

The prevalence vastly differed based on geographic regions. The pooled prevalence of RLS in Europe was 20.4%–22% (15, 169), and in Western Pacific was 14% (15). Whereas, the pooled prevalence of the Region of Americas was 20% and Eastern Mediterranean was 30% (15). There is little data on Southeast Asia and Africa; however, RLS seems to be more common in White and Asian women compared to Black women (175). A study conducted on a sample of pregnant Nigerian women found the prevalence of RLS based on a subjective definition of RLS to be 0% (173). Whereas within pregnancies in Southeast Asia, the prevalence was 19.9% in Japan (167), and 10.4% in Taiwan (171). The differences in RLS incidence among race was studied by Na et al. (175), which corroborates the above data. The cumulative incidence of new onset of RLS in their cohort of 2,704 healthy pregnant people was 18.1% on average, and 21.1% for Asian, 20.3% for White, 17.1% for Hispanic, and 15.4% for Black women (175).

### Risk factors

Several risk factors are associated with developing RLS in pregnancy. Maternal age of 25 years and older (169, 175), nulliparity (175), and advanced gestational age (23, 176), are risk factors for RLS. Sociodemographic factors associated with RLS include lower educational status (177), living in a joint family (177), or being of Hispanic descent (175). Obesity (178) and total skin folds of the subscapular and triceps site (independent of BMI) were also associated with RLS (175).

Dietary factors such as coffee consumption (171), vitamin deficiency (179), zinc and magnesium deficiency (180), and iron deficiency (164, 170, 181), have been identified as risk factors. Along with iron deficiency, anemia (175), low total iron binding capacity (178), low ferritin levels (176), low folate levels (170), and low hemoglobin levels (177, 179, 182), were cited factors also associated with greater severity of symptoms. Genetics may play a role in RLS development in pregnancy including history of RLS in previous pregnancies and family history (130, 136, 142, 150, 152), which increases risk. Family history or childhood history of growing pains may also be predictors (168).

### Associations/outcomes

#### Maternal

The literature has identified associations between RLS in pregnancy and comorbid conditions. Maternal mood is associated with RLS in pregnancy, with depression being most common (170). Wesström et al. (172) found those who presented with moderate or severe RLS prior to pregnancy were at increased risk of antenatal and postnatal depression. People with postpartum depressive symptoms have higher prevalence of RLS in the last trimester of pregnancy (42). Similarly, a positive association with RLS severity and anxiety symptom severity has been observed (180).

Interestingly, peptic ulcer disease and thyroid disease were associated with RLS in pregnancy (124). Pregnant people with RLS were also more likely to have other sleep problems including night cramps, excessive daytime sleepiness, sleep-wake disturbances, poor sleep quality, snoring, early awakening and insomnia (161, 166, 167, 169, 178, 179, 183). In particular, there was a dose-response relationship found between the RLS symptom frequency and sleep-wake disturbances (i.e., poor sleep quality, excessive daytime sleepiness) (166). Similarly, other studies have shown that RLS in pregnancy is associated with severe sleep disorders (166, 180). Snoring is also correlated with a high prevalence of RLS across all trimesters (183).

HDP are also related to RLS. In particular, preeclampsia was more frequent in a population of pregnant people with RLS (184). There may be a link between severity of HDP and RLS, although this is supported by weak evidence (174, 185, 186). Furthermore, the presence of pregnancy-induced hypertension or chronic hypertension are associated with RLS (186, 187). A personal history of pregnancy-induced hypertension was also found to be associated with RLS (187). In terms of delivery outcomes, those with RLS were also more likely to have a cesarean delivery compared to those without RLS in pregnancy (161).

#### Fetal

Some adverse fetal outcomes may be associated with RLS in pregnancy including threatened spontaneous abortion, threatened preterm labor, and intrauterine growth restriction (164). Furthermore, leg movement score, a measure of RLS, has a significant association with neonatal birthweight and gestational age at birth (174). People with RLS were also found to be more likely to have infants with higher fetal biparietal diameter and femur length (180).

## Insomnia

Insomnia is a sleep disturbance manifesting as a symptom/disorder in the general population, and becomes more common in pregnancy. Our search yielded 29 papers that evaluated this concept.

### Characterization

According to the DSM-5, insomnia is defined as dissatisfaction with sleep quantity/quality causing distress/impairment in areas of functioning that is accompanied by symptoms such as difficulty initiating sleep, difficulty maintaining sleep, or early morning awakening with inability to return to sleep (188). This disturbance must occur at least three nights a week, exists even with opportunities for sleep, and cannot be attributable to another condition/substance in order to be considered primary insomnia (21, 188). Pregnancy is a vulnerable period in which insomnia, can often manifest or become exacerbated as a result of new physiologic stressors. This can be attributed to the hormonal, anatomical and psychosocial changes associated with pregnancy (189), among other unknown factors. For example, physical pain or reduced bladder capacity are stressors that appear in pregnancy that may influence the development of insomnia (21). Restless sleep, leg cramping, lower back pain, or nightmares may also be causes during pregnancy (190, 191).

There are several instruments used to characterize insomnia such as the insomnia severity index, women’s health initiative insomnia rating scale, among others. In general, there is worsening of self-reported insomnia scores as pregnancy progresses (65). Pregnant people also complain of nocturnal awakening, sleep-onset difficulties, and poor sleep quality (192). Objectively, pregnant women are more likely to have lower sleep efficiency, decreased REM sleep time, increased stage 1 sleep (“light sleep”), increased movement while asleep, and spend longer time in bed (190, 193). The shortest REM sleep time was found between 35–38 weeks of pregnancy (190). Self-reported insomnia scores and objective measures improved postpartum (190, 194).

### Prevalence

A consensus has been developed from various population-based studies that almost one-third of general society experiences insomnia symptoms while approximately 10% report insomnia disorder (195). Insomnia prevalence is higher in pregnancy than in the general population; however, the cited prevalence is variable. Variability was dependent on the sample size, geographic location, and diagnostic instrument used to define insomnia. A meta-analysis by Sedov et al. (21) found the overall prevalence of insomnia symptoms during pregnancy to be 38.2%. Other studies have observed higher prevalence rates, around 40%–63% (191, 193, 194, 196–198). A Finland-based study that explored the prevalence of insomnia symptoms in a sample of 1,667 mothers and 1,498 fathers found worsening sleep quality in not only pregnant women but also male partners (192).

The literature largely supports an increased prevalence of insomnia throughout pregnancy, with improvement after birth (189, 194). Specifically, an increase is noted from the first trimester to the second trimester (199). The prevalence of insomnia in the first trimester is 25.3%, which jumps to 27.2% in the second trimester (21). This further increases in the third trimester (200). The prevalence of insomnia in the third trimester based on an updated meta-analysis of 10 studies was 42.4% (22). Kızılırmak et al. (196) found the risk of insomnia to be 2.03× higher in the third trimester compared to the first and second trimester. Possible contributory factors for this considerable increase in the third trimester could be frequent rising to void, not finding a comfortable sleeping position, musculoskeletal discomfort, and restless legs.

Despite several studies highlighting reduction in insomnia in the postpartum period, not all studies agree. Within Sivertsen’s et al. (201) cohort of 1,480 pregnant people, the prevalence of insomnia was stable at 32 weeks’ gestation, 8 weeks postpartum, and remained high at 2 years postpartum. This may be due to rapidly changing patterns of sleep associated with care of a newborn, leading to persistence of insomnia (201). However, given that approximately 16 weeks (4 months) occurred between their first two measurements, one explanation for this discrepancy could be that the prevalence of insomnia continued to increase after 32 weeks’ gestation and peaked at a rate higher than the rates that they saw at these two time points (32 weeks’ gestation and 8 weeks postpartum) and interpreted to be stable (201).

### Risk factors

Several risks have been associated with an increased insomnia risk in pregnancy, which can be broadly categorized as maternal factors, lifestyle, and comorbidities. Older age and lower educational level are risk factors for insomnia (189, 196, 202). Women who are 20 years and older have a 2.19 greater odds than younger women for developing insomnia during pregnancy (196). Higher blood pressure or high pre-pregnancy BMI also increase risk (189, 196, 200). Pregnancies affected by obesity have an increased odds of developing insomnia in the third trimester (aOR 2.3) (189, 196, 200). Lifestyle habits may impact insomnia development by inducing physiological or psychological stress. Smoking and eating at night both increase the risk of insomnia (189, 191, 202). A protective factor seems to be physical activity as moderate physical activity decreases the odds of insomnia in the third trimester (aOR 0.65) (200).

Furthermore, a previous history of insomnia significantly increases risk of development in pregnancy. Román-Gálvez et al. (200) found pre-gestational insomnia to be a determinant of first trimester insomnia (aOR 12.50). Those who had insomnia earlier in pregnancy were more likely to have insomnia in the second trimester (aOR 4.21) and third trimester (aOR 4.43) (200). Having a psychiatric condition is also a risk factor, specifically depression (191, 196). Within a sample of *N* = 257 perinatal patients who sought psychiatric outpatient treatment, more than 50% reported moderately severe insomnia symptoms and 12% reported severe insomnia symptoms (203). Risk of insomnia has been found to be more than two-fold higher for those who had depression than those who did not (196). Kalmbach et al. (197) found pregnant people with depression are at a greater risk for sleep-onset insomnia (OR 2.80) and sleep-maintenance insomnia (OR 6.50). Conversely, despite being highly associated, insomnia in pregnancy was not found to be a risk factor for postpartum depression (193, 194), which is counterintuitive given the strong link between poor subjective sleep quality and postpartum depression (204, 205).

### Associations/outcomes

#### Maternal

There is extensive literature exploring the associations between insomnia and maternal outcomes. Individual factors that increase physiological vulnerability to insomnia in pregnancy include older age and parity (65). Interestingly, personality may also influence sleep patterns. Insomnia during pregnancy was associated with specific personality traits, such as neuroticism and agreeableness (206). Maternal insomnia affects quality of life for pregnant people and has been found to lead to decreases in physical health, social relationships, and environmental health (202). Regarding physical health, a longitudinal cohort study of 1,480 women found a 2.8-fold increased odds of reporting high levels of postpartum bodily pain for those who were defined as having chronic severe insomnia (207). Other studies have found associations between insomnia and pelvic girdle pain and/or lower back pain (198). Preeclampsia has also been linked with insomnia—severe preeclampsia has been associated with increased severity (208).

Associations between maternal insomnia and psychiatric disorders are well established. A meta-analysis of 9 studies found a significant relationship between insomnia and perinatal depressive symptoms (209). This is in alignment with a large body of evidence and is of no surprise as sleep disturbance is one of the criterion for depression (194, 202, 210). Baseline insomnia symptoms pre-pregnancy are associated with depressive symptoms at each point in pregnancy (65). In conjunction with high nocturnal rumination or nocturnal cognitive arousal, pregnant people who experience insomnia are also more likely to endorse high rates of depression and suicidal ideation (197, 211). Insomnia symptoms were not just associated with depressive symptoms in pregnant women (aOR 3.9) but also among male partners (aOR 1.9) (192). Similarly, there is a strong relationship between insomnia in pregnancy, anxiety, and stress (181, 183, 184). Osnes et al. (212) found insomnia to be significantly associated with postpartum anxiety symptoms. They also found mid-pregnancy insomnia to be significantly associated with concurrent and postpartum anxiety among 530 participants (213).

Other intrinsic factors such as maternal sleep reactivity and nocturnal rumination are associated with insomnia (214). Nocturnal rumination is strongly associated with sleep onset difficulties rather than sleep maintenance (197). Conversely, mindfulness is negatively associated with insomnia, with those who practiced mindfulness reporting less insomnia symptoms (215).

#### Fetal

There were limited results on fetal outcomes in this context. However, there is a link between insomnia and risk of PTB. Based on a cohort of approximately three million California women, the odds of PTB (defined as birth before 37 weeks’ gestation) was 1.3x compared to a reference population (95% CI, 1.0–1.7) (148).

## Circadian dysrhythmia

Our analysis would be incomplete without highlighting the potential role that disruptions in circadian rhythm may have on reproduction and pregnancy. Our search identified 27 studies relating to this issue.

It has long been hypothesized that sleep disruption can alter hormones and impact fertility and pregnancy (5, 216). Known as the sleep hormone (217), melatonin is a fundamental regulator of the circadian rhythm. Owing to its ability to upregulate antioxidant activity (218), melatonin has links to fertility (219–221), appears neuroprotective in the perinatal period (222, 223), and may prolong the latency from preeclampsia diagnosis to delivery (224–228). That said, melatonin’s interactions with pregnancy present a significant research gap. Beyond melatonin, however, reproductive function in humans is regulated by sex hormones that are synthesized, secreted, and metabolized in a way that aligns with the sleep pattern and circadian timing of the body (229). The ill effects of sleep disturbances act primarily *via* a stress response mediated by activation of the hypothalamic-pituitary-adrenal and dysregulation of the hypothalamic-pituitary-ovarian axes. Perturbations in sleep drive overproduction of corticosterone and alter gonadotropin and sex steroid secretion, which leads to anovulation, amenorrhea, failed embryo implantation, and infertility (6, 229). Circadian disruption occurs with shift work, jet lag, daylight savings time, and chronobiology, and is associated with poor fertility and sub-optimal early pregnancy outcomes (5).

As reviewed by Willis et al. (6), several studies have demonstrated that poor sleep quality is linked to delayed time to pregnancy and infertility. The molecular mechanisms associated with circadian dysrhythmia are driven by alterations in the expression of circadian rhythm-regulating circadian locomotor output cycles kaput (CLOCK) genes and are reviewed in detail elsewhere (5, 6, 229). The overall impact of circadian rhythm on reproductive function should not be ignored. Gaining a better understanding of the mechanisms at play, including modifiable human behaviors, can enable the design of interventions to optimize reproductive outcomes.

### Circadian dysrhythmia: associations and outcomes

The circadian clock system regulates the sleep-wake cycle, controls feeding-fasting, and regulates reproductive processes in humans (230). Quantifying the prevalence of circadian dysrhythmia is challenging given the heterogeneity in study design, populations assessed, the varying measurement tools used, study type, and sample sizes.

As previously highlighted, sleep problems during pregnancy can increase the risk of adverse outcomes. In 2017, Merikanto et al. (231) studied 1,653 pregnant women from the Finnish CHILD-SLEEP Birth Cohort to assess the impact of chronotypes and lifestyle habits. Overall, it was demonstrated that women with a predilection for evening activities (evening chronotypes) had more sleep problems, trouble falling asleep, poorer sleep quality, daily fatigue, were more often smokers, and reported poorer general health than morning chronotypes. Similarly, in a controlled study, Liu et al. (232) showed that long exposure to electronic screens before nocturnal sleep in pregnancy is associated with HDP. When handheld devices were used for entertainment purposes only, the risk of HDP increased.

Later sleep timing and circadian preference have been linked to worse health outcomes across multiple domains including mood disorders, substance use, impulse control, and cognitive function. Obeysekare et al. (233) examined whether sleep timing during the third trimester of pregnancy could predict postpartum symptoms of mania, depression, and obsessive-compulsive disorder in a sample of 51 women with a previous, but inactive, episode of unipolar and bipolar depression. In this study, patients were assessed for symptoms and sleep was quantified with wrist actigraphy at 33 weeks’ gestation and 2, 6, and 16 weeks postpartum. Overall, a delay in sleep timing in this sample of at-risk women was associated with more symptoms of mania, depression, and obsessive-compulsive disorder in the postpartum period and suggests that sleep timing may be a modifiable risk factor for postpartum depression.

These results align with those of Meliska et al. (234) who built on previous research suggesting that a patient’s mood can vary in a seasonal manner as a result of light-modulated alterations in chronobiologic markers such as melatonin and cortisol. In this small study in depressed antepartum patients, they found that depression severity during pregnancy may be elevated in association with seasonally-related phase changes in melatonin and cortisol timing. They also demonstrated a reduction in melatonin quantity that occurred in patients with depression when compared to healthy controls. Overall, Meliska’s et al. study suggests that patients with antepartum depression may be more susceptible to the physiological changes induced by seasonally longer nights, highlighting the need for more research in this area.

Recently, sleep disturbance has been identified as a risk factor for GDM. Short sleep has also been linked to chrono-nutritional disorders including increased meal frequency and dietary content related to sleep-wake cycles, disorders of sleep quality, and abnormal weight gain as a result of sleep restriction and time of eating. Thus, Messika et al. (235) completed a randomized controlled trial of the impact of a chrono-nutritional and sleep hygiene intervention on maternal glycemic control and the proportion of LGA fetuses in pregnancies with GDM. Although the intervention had no impact on the risk of LGA, it reduced the proportion of women with suboptimal glycemic control. This effect was largely driven by a reduction in carbohydrate intake in the evening and persisted after adjustment for known confounders. These results further support a relationship between poor sleep and impaired glucose control and also provide support that an intervention focussing on sleep hygiene may improve glycemic control.

### Shift work in preconception and pregnancy: associations and outcomes

Several studies have suggested that sleep disturbance from shift work, long work hours, and erratic schedules may alter one’s hormonal milieu contributing to increased miscarriage risk, altered fetal growth, and pregnancy complications. However, the evidence is conflicting with some studies showing little or no effect (236–241), and others reporting negative effects (242–248). Overall, it remains unclear if varying work schedules affect pregnancy risk or fetal well-being. The speculated associations appear to stem from studies that attribute maternal hormonal disturbances to sleep deprivation or circadian rhythm disruption and link this to impaired fetal growth and pregnancy complications.

#### Studies showing little-to-no effect

Zhu et al. (236) showed that shift work during pregnancy had little effect on indicators of fetal growth and that prolonged night shift work may prolong pregnancy and reduce fetal growth—an association that was more pronounced in industrial workers. They examined data from the Danish National Birth Cohort and used regression analysis to estimate the effect of shift work on the duration of pregnancy and birth weight from 1998 to 2001 and included only singletons. Of the 32,465 women with daytime work, 1,038 women with fixed evening work, 400 women with fixed night work, 3,137 women with rotating shift work (without night), and 3,197 women with rotating shift work (with night), during the first and second trimesters of pregnancy, they did not show an association between gestational age at birth or birth weight at term between any of the groups. However, they did highlight a significant relationship between fixed night work and likelihood of postterm birth (OR 1.35) and fixed evening work and risk of LBW at term (OR 1.80). They concluded that the relationship between shift work during pregnancy and fetal outcomes appears to be limited.

The limited effect of shift work on negative pregnancy outcomes has also been the focus of several reviews. In a systematic review of 22 epidemiological studies, Schlünssen et al. (238) observed no convincing associations between rotating shift work or fixed night shift work and risk of spontaneous abortion, PTB, or LBW. However, there was some support for fixed night shifts and stillbirth. In a subsequent analysis, they suggested that if fixed night work was avoided in their cohort, 7 stillbirths a year could be avoided, leading them to discourage this type of work schedule in pregnancy.

Bonzini et al. (239) further addressed the impact of varying work schedules and circadian disruption arising from sleep deprivation on fetal wellbeing. In this study, they aimed to reconcile previous meta-analyses showing a small adverse effect of shift work on PTB. Of the 23 relevant studies included, they observed a small effect of shift work for PTB (RR 1.03), LBW (RR 1.27), and SGA (RR 1.12). Their results are in line with previous preliminary studies and reviews suggesting that the overall risk of poor birth outcomes resulting from shift work in pregnancy is small.

Furthermore, Bonde et al. (240) aimed to reconcile previous conflicting reports examining the relationship between shift work and an increased risk of miscarriage. In their systematic review of 30 studies from 1966–2012, looking at the RR of miscarriage with occupational exposure and excluding studies most likely to be biased, they showed that working fixed nights was associated with a moderate increased risk of miscarriage (RR 1.51). This association was attenuated when only high quality studies were included. Thus, they concluded that while a small association exists, it does not warrant mandatory restrictions in relation to shift work. Nonetheless, they did suggest that people with high risk pregnancies should receive individualized counseling vis-à-vis shift work and other occupational exposures.

Maternal sleep disturbance and circadian disruption has recently garnered attention for its potential impact on downstream child health. Ramlau-Hansen et al. (237) aimed to assess the associations between shift work during pregnancy and male offspring’s semen quality and quantity and levels of reproductive hormones in a small pilot study. Using data from the Danish pregnancy cohort, 327 sons aged 18–21 years were evaluated, and semen and blood samples were taken. Maternal data was collected from questionnaires that included information on shift work. Of the 278 sons with data available, 15% were exposed to maternal shift work. Relative to the unexposed group, those exposed to maternal shift work had a 30% lower percentage of normal sperm morphology; however, no associations were found with all other semen parameters and reproductive hormones, which suggests a limited intergenerational effect. Notwithstanding, the results of this small experimental study must be taken in context and are more hypothesis generating.

Similarly, the impact of night shift work before and during pregnancy on offspring weight trajectory was assessed by Strohmaier et al. (241) who postulated that maternal circadian disruption may have a lasting impact on child metabolism. In this retrospective cohort study, 4,000 children and their mothers were included. Pre-pregnancy night shift work was modestly associated with child overweight and obesity (RR 1.11). However, longer duration of rotating shift work was not associated with any child weight trajectory, nor was child weight associated with night shift work, regardless of shift frequency. Overall, it appears that night shift work before and during pregnancy had little, if any, impact on offspring weight outcomes.

#### Studies showing detrimental effects

While the aforementioned studies examining the impact of shift work and circadian dysregulation demonstrated a limited impact on pregnancy and downstream child outcomes, several studies have reported relationships that warrant consideration.

Zhu et al. (244) reported that fixed night shift work was associated with fetal loss. Using the Danish National Birth Cohort, they examined 33,694 pregnancies of daytime workers and 8,075 pregnancies of shift workers and showed that fixed night work was associated with fetal loss (HR 1.85) and that this relationship was not evident for other types of shift work nor self-reported job stress.

Similarly, Feodor Nilsson et al. (245) sought to identify modifiable risk factors for miscarriage before 22 weeks and estimated the preventable proportion of miscarriages that could be attributed to these risks. In this nationwide observational follow-up study using the Danish National Birth Cohort, they included 91,427 pregnancies. Of the 88,373 pregnancies that had full information on all covariates, the potentially modifiable preconception risk factors associated with increased miscarriage risk were age >30 years, underweight, and obesity. During pregnancy the modifiable risk factors were alcohol consumption, lifting of >20 kg daily, and night work. They also combined several of these modifiable risk factors to determine the potential impact on miscarriage reduction and showed that approximately 25% of miscarriages could be prevented if all these risk factors were reduced to low risk levels. Overall, this large population based study strongly suggests that there are several potentially modifiable risk factors for miscarriage prevention, including sleep; however, age at conception and alcohol consumption were the most important.

More recently, in 2020, Suzumori et al. (248) assessed the impact of the frequency of night shifts during pregnancy on adverse outcomes. In a prospective cohort study conducted in 15 regions in Japan, they included 99,744 singleton pregnancies and linked their medical records to the parturients’ working hours and frequencies of night shifts during the first and second/third trimesters using a self-administered questionnaire. When comparing those who worked during pregnancy to those who did not, those who worked had significantly increased aOR of threatened miscarriage (aOR 1.47) and of threatened PTL (aOR 1.63). The association of hours worked with HDP was exacerbated when women worked ≥36 h per week with night shifts (aOR 2.02). They were also shown to have increased likelihood for vacuum/forceps birth (aOR 1.34) at ≥36 h with or without night shifts, and for SGA infants (aOR 1.32) at ≥46 h with night shifts. Lower aOR’s were observed for GDM and meconium-stained amniotic fluid in those working without night shifts. Taken together, compared to those who did not work, those who worked during pregnancy had an increased risk of miscarriage and threatened PTL, and working long hours exacerbated pregnancy risks, especially with night shifts.

In 2019, Wei et al. (247) studied the associations between maternal shift work during pregnancy and infant neurodevelopmental outcomes using the Taiwan Birth Cohort Study. In this large retrospective study, child neurodevelopment and exposures were assessed by home interviews with questionnaires at 6 and 18 months of age. A total of 5,637 term singletons were included, and a sub-population of 2,098 cases were selected in a propensity-score matched cohort. Overall, it was shown that persistent maternal shift work during pregnancy was associated with increased risk of delayed gross-motor (aOR 1.36 for walking steadily), fine-motor (aOR 1.39 for scribbling), and social neurodevelopmental milestones (aOR 1.35 for coming when called upon). The negative relationships between maternal shift work and delayed gross-motor and social development were also identified in the propensity-score-matched sub-cohort. Although the underlying mechanisms driving this association remain to be established, this study suggests that maternal shift work may have ill-effects on early childhood developmental milestones and these may persist into the future.

## Discussion

Sleep-disordered breathing (SDB) emerges as a prominent sleep disorder during pregnancy. Physiological changes in the respiratory system and sleep contribute to an increased risk of SDB. Prevalence reports differ from clinical diagnoses, with higher commonality in the former and rarity in the latter. SDB symptoms and severity progressively worsen as pregnancy advances, peaking after childbirth when progesterone levels decline. Many SDB risk factors are well-established but non-modifiable (e.g., maternal age) or have limited modifiability (e.g., GWG). SDB is consistently associated with adverse maternal and fetal outcomes during and beyond pregnancy, though causality remains unclear. It’s important to consider comorbidities and confounding factors when studying SDB in pregnancy, particularly with obesity, diabetes, and hypertensive disorders. These conditions impact SDB severity, and SDB reciprocally affects them through various physiological mechanisms. Pregnancy-onset SDB is distinct from chronic SDB and requires separate analysis. While most cases of pregnancy-related SDB are mild, moderate and severe cases also exist and should be categorized accordingly in research.

Significant progress has been made in understanding SDB in pregnancy, thanks to the dedication of researchers, clinicians, and patients worldwide. However, knowledge gaps persist, potentially due to the complexity of SDB in pregnancy or limitations in knowledge and methodology. Heterogeneity in findings can be attributed to variations in measurement tools, both subjective and objective. Longitudinal studies exploring SDB throughout the reproductive cycle and beyond the first postpartum year are limited, highlighting a major research gap. Long-term follow-up is crucial, especially considering the potential link between SDB in pregnancy and future cardiovascular disease. Future research should prioritize the use of validated and objective measures across the reproductive cycle, extending into middle age, menopause, and beyond to better comprehend the relationship and its lifelong impact on women’s health.

Our search revealed a significant body of literature on RLS, a transient occurrence in pregnancy influenced by hormonal changes. RLS symptom severity tends to increase as gestation progresses, peaking in the third trimester and resolving after delivery. However, variability in defining RLS and differences in study populations result in inconsistent prevalence rates. Geographic variations exist, but limited data on regions like Southeast Asia and Africa prevent definitive conclusions. Iron deficiency, genetic predisposition, and personal/family history of RLS are established risk factors. RLS is associated with maternal comorbidities, including other sleep disorders and psychiatric conditions, highlighting the interplay between pregnancy, sleep, and mental health. Measurement of RLS poses discrepancies among studies, emphasizing the need for standardized, validated measures. Additionally, research should explore understudied geographic regions to elucidate the role of ethnic background as a risk factor.

Insomnia, another common sleep disorder identified in our review, is influenced by pregnancy-specific stressors and either worsens pre-existing insomnia or leads to new-onset symptoms. The prevalence of insomnia during pregnancy was generally higher than in the general population, increasing with advancing gestational age. However, data on postpartum insomnia rates were conflicting. It’s important to acknowledge that insomnia assessment relied on self-reported measures, contributing to heterogeneity and highlighting measurement challenges once again. The literature identifies several modifiable risk factors for insomnia. Additionally, insomnia showed a strong association with maternal psychiatric disorders, such as depression and anxiety. Notably, there was a lack of research on fetal outcomes in both RLS and insomnia, revealing a significant knowledge gap in the field of sleep disorders during pregnancy and emphasizing the need for future research in this area. Catastrophizing insomnia, personality traits, and sleep reactivity, which are among the main factors perpetuating insomnia, should also be investigated during pregnancy and the postpartum period (195, 249–251).

Poor sleep is linked to adverse health outcomes such as obesity, type 2 diabetes, cardiovascular disease, and depression. The role of circadian rhythms in reproductive function is gaining interest (6, 229). In reproductive epidemiology, short sleep is associated with higher risk of adverse pregnancy outcomes like GDM, stillbirth, PTB, and LBW (26, 252). Researchers are now focusing on quantifying sleep exposures beyond occupational hazards and shift work, revealing associations between menstrual cycle irregularity, subfertility/infertility, and sleep disorders (6). The relationship between circadian dysrhythmia and poor reproductive outcomes involves complex factors including genetic predisposition, dysregulation of hormone axes, insulin resistance, and oxidative stress (6, 216). Sleep disturbances during pregnancy are common, but whether improving short or disordered sleep can enhance reproductive and obstetrical outcomes is a topic still in its infancy.

In future research, it is important to avoid oversimplification of sleep behavior by solely examining sleep duration. Instead, comprehensive assessments should incorporate measures of sleep quality, optimal timing of sleep, and the presence of sleep disorders (253). While standardized questionnaires like the PSQI have value at a population level, reliance on self-reported measures should be minimized. Objective measures such as PSG, home sleep apnea testing, and actigraphy should be utilized, alongside the inclusion of relevant confounding variables that affect a person’s sleep hygiene during their reproductive years. These variables may include their partner’s sleep status, comorbidities like obesity and depression, and the involvement of their partner in infertility studies. By taking these steps, we can begin unraveling the complexity of sleep’s impact, quantifying its relationship with reproductive outcomes, and ultimately enhancing the health of women, their infants, and future generations.

## Author contributions

AK and ZF: idea development, title/abstract/full-text search, data extraction, and manuscript editing. PE: literature search, title/abstract/full-text search, data extraction, and manuscript editing. CJ and SH: manuscript editing. All authors contributed to the article and approved the submitted version.

## Conflict of interest

AK is the majority shareholder and the volunteer (unpaid) Chief Executive Officer of Shiphrah Biomedical Inc., which is a startup company with an interest in sleep in pregnancy research and development.

The remaining authors declare that the research was conducted in the absence of any commercial or financial relationships that could be construed as a potential conflict of interest.

## Publisher’s note

All claims expressed in this article are solely those of the authors and do not necessarily represent those of their affiliated organizations, or those of the publisher, the editors and the reviewers. Any product that may be evaluated in this article, or claim that may be made by its manufacturer, is not guaranteed or endorsed by the publisher.
